# Assessment of Various Mitigation Strategies of Alkali-Silica Reactions in Concrete Using Accelerated Mortar Test

**DOI:** 10.3390/ma17205124

**Published:** 2024-10-21

**Authors:** Abdullah Almakrab, Mohamed T. Elshazli, Ahmed Ibrahim, Yasser A. Khalifa

**Affiliations:** 1 Department of Civil and Environmental Engineering, University of Idaho, Moscow, ID 83843, USA; a.almakrab@rcu.gov.sa (A.A.); y.khalifa@mtc.edu.eg (Y.A.K.); 2 Department of Civil and Environmental Engineering, University of Missouri, Columbia, MO 65202, USA; elshazlim@missouri.edu; 3 Department of Civil Engineering, Military Technical College, Cairo 11865, Egypt

**Keywords:** alkali-silica reaction, supplementary cementitious materials, accelerated mortar test, lithium, metakaolin, NewCem Plus

## Abstract

The widespread use of reinforced concrete continues to face challenges, particularly in mitigating alkali-silica reaction (ASR), due to its detrimental effects on concrete strength and durability. This paper investigates the effectiveness of using binary supplementary cementitious materials (SCMs) in mitigating ASR by incorporating metakaolin (MK) and waste glass powder (GP) as partial replacements for cement. Additionally, the potential of a new cement product, “NewCem Plus” (NCM), along with the use of basalt fibers and lithium, was evaluated through a 14-day accelerated mortar bar test following the ASTM C1260. This study also assessed concrete’s properties such as its compressive strength and workability using the flow test. The results indicated that MK was effective, reducing expansion by 79%, 84%, and 88% with 10%, 20%, and 30% cement replacement, respectively, compared to the control mixture. On the other hand, GP showed a more modest reduction in expansion, with 10%, 20%, and 30% replacement levels reducing expansion by 20%, 43%, and 75%, respectively. Furthermore, the addition of lithium to MK significantly mitigated ASR, reducing expansion below the ASTM threshold. However, mixtures containing NewCem Plus, lithium, and basalt fibers showed minimal impact on ASR reduction. These findings underscore the viability of using binary or ternary blends of SCMs to mitigate ASR in concrete, encouraging their adoption in future concrete applications.

## 1. Introduction

Concrete is an essential material in most construction activities and is known to be the most widely used construction material globally [1,2,3], primarily due to its excellent strength and the ease with which it can be produced from abundant, natural materials. Despite its widespread use, concrete faces challenges arising from environmental conditions. Factors such as temperature fluctuations and moisture variations can cause concrete elements to crack and accelerate other forms of deterioration. One of the most significant durability concerns for concrete structures across various applications is the alkali-silica reaction (ASR). ASR occurs due to the interaction between amorphous silica present in some natural aggregates and the hydroxyl ions in cement [4,5,6]. This reaction can take years to become visible and causes significant structural damage over time, and the early diagnosis of ASR is complicated; therefore, mitigating ASR using various alternative approaches has become critical. Furthermore, current methods for addressing or preventing ASR generally do not create permanent solutions, underscoring the need for continuous assessment and more effective long-term solutions.

ASR is recognized to be the main factor contributing to the corrosion-induced degradation of concrete roadway infrastructure in the United States [7,8]. The ASR produces a gel with a hygroscopic nature, that has a strong capacity to absorb water from the surrounding environment. This absorption leads to the swelling of the gel when water is present in the pore solution, resulting in internal expansion and eventually causing fractures within the concrete [5,9,10]. This damaging reaction manifests through pop-outs, surface discolorations, extrusions, and cracking, all of which contribute to the gradual degradation of the structure [8,11,12]. To address this issue, extensive research has been directed toward identifying effective mitigation strategies, with one of the most promising approaches being the use of supplementary cementitious materials (SCMs) [4,13]. These materials, when incorporated into concrete mixtures, have been shown to reduce the potential for ASR by modifying the chemical composition and reactivity of the cement. Moreover, the use of new waste by-products and various fiber types holds potential for further exploration in ASR mitigation. To ensure the reliability and speed of these approaches, accelerated testing methods, such as those outlined in the ASTM C1260, offer a quick and practical means of evaluating the efficacy of SCMs and other materials against ASR [14,15].

The body of literature addressing the mitigation of ASR is extensive, with new experiments and research being conducted annually. In recent years, several SCMs have been extensively studied, including fly ash, silica fume, blast furnace slag, glass powder, and metakaolin [16,17,18,19,20,21]. These studies have shown that SCMs can effectively inhibit ASR, though the degree of effectiveness highly depends on the chemical properties and the proportion of the SCMs used. Many studies have also observed that ASR stops when the internal relative humidity of concrete remains below 80–85% [22]. Helmuth et al. (1993) found that thin concrete elements are less likely to be affected by ASR [22]. However, the risk posed by ASR increases significantly in larger concrete members, especially in arid regions, where high internal humidity levels are often sustained [23].

Surface film coatings, such as water-repellent agents and polyurethane, including water-based silicates, have proven ineffective for preventing water penetration over extended periods. Hobbs (1988) [24] investigated cracked concrete piers supporting an expressway in Japan. These piers were repaired seven years after construction by filling the cracks with pressure-injected epoxy resin. The piers were then coated with saline impregnation or epoxy resin, and a topcoat of polymer cement paste was applied. However, his study concluded that this method was not as effective as initially anticipated. The repair only delayed the inevitable deterioration, as cracks reappeared after several years of exposure. This finding indicated that crack injection, intended to remove moisture and apply surface coatings, yielded limited long-term results. Charlwood and Solymar (1995) [25] found that ASR propagation could be temporarily mitigated by sealing construction joints or macrocracks with cement epoxy or grout resins, typically applied just before the use of a waterproof sealant. However, once a structure shows signs of ASR, the destructive reaction cannot be permanently halted. ASR tends to reoccur once moisture is reabsorbed by the concrete, reactivating the reaction [26]. Flexible grouts, as opposed to epoxy resins, have demonstrated better success in preventing leakage through cracks or gaps, particularly when ASR is still progressing [27,28].

Farny & Kerkhoff (2007) [1] emphasized the importance of waterproofing as a critical method for reducing ASR in concrete. Similarly, De Beauchamp (1995) [29] reported the successful application of waterproof membranes, such as polyvinyl chloride (PVC), to the surface of concrete dams, which provided effective protection against water infiltration into concrete structures. Recent research has also examined the use of basalt fibers for ASR mitigation, with Lipatov et al. (2015) [9] and Guo et al. (2018) [11] presenting findings that demonstrated a noticeable improvement in both tensile and flexural strengths when basalt fibers were incorporated into concrete, offering an economical solution. On the other hand, Haddad et al. (2004) [30] explored the use of steel fibers coated with brass, concluding that while these fibers did not contribute to ASR mitigation, they did result in enhanced tensile strength in concrete. These findings highlight the potential benefits of fiber reinforcement in improving the mechanical properties of concrete, even if their effects on ASR mitigation vary.

Grounded glass, a waste by-product, has recently been utilized to reduce ASR. Glass waste is one of the most common solid waste products globally [31], and it presents a significant environmental hazard that requires urgent solutions [32,33]. Recycling waste products, including glass, is critical due to the large volumes produced, the high CO_2_ emissions from cement plants, and other greenhouse gases that contribute to environmental degradation, human health risk, and climate change [27,28,32,34,35,36,37,38]. Grounded glass has a chemical composition similar to regular Portland cement, making it a suitable substitute for cement or sand in concrete mixtures [39,40,41,42,43]. Numerous studies have examined the use of waste glass powder, either as a partial replacement for cement or aggregate, as a promising alternative for ASR mitigation [41,44,45,46,47,48]. The role of microparticle size in waste glass for mitigating ASR has been highlighted in studies by Kara De Meijer (2014) [8] and Ke, et al. (2018) [7]. However, an excessive amount of waste glass powder, particularly when combined with Class C fly ash, can have undesirable effects, potentially increasing ASR [39,46]. Similarly, MK has been shown to mitigate ASR, but the overall characteristics of concrete, such as its workability and strength, may be negatively impacted when high amounts of silica fume or MK are used [1,49]. Additionally, studies have found that excessive amounts of fly ash or slag can detrimentally affect both the workability and early strength of concrete [50,51]. These findings suggest that while waste glass and other SCMs offer benefits for ASR mitigation, their optimal usage and proportioning are crucial for maintaining the desired performance of concrete.

These considerations emphasize the importance of maintaining a balanced approach when incorporating pozzolans into concrete mixes. It has been proposed that the use of two or more SCMs in combination can mitigate the effects of ASR while preserving the essential technical properties of concrete. A particularly promising method for reducing ASR and enhancing other concrete properties is the use of ternary blends in pozzolan-containing mixtures [1,52]. Research has shown that ternary blends are efficient and effective than binary blends in improving the mechanical, rheological, and durability performance of concrete [53,54,55,56]. However, relatively few studies have focused on the ternary use of glass powder in conjunction with other pozzolans. The effectiveness of ternary mixes containing fine glass powder in mitigating ASR was explored by Afshinnia and Rangaraju (2015) [54]. Their research found that the use of ternary mixes with glass powder, as opposed to binary mixtures, significantly reduced ASR expansion. Afshinnia and Rangaraju (2015) also investigated the properties of binary and ternary cement pastes containing glass powder, though the range of glass powder blends examined was limited. Consequently, further comparative studies are needed to fully assess the performance of ternary mixtures incorporating glass powder at various proportions, to optimize their use for ASR mitigation.

Phosphogypsum, another by-product of the phosphate fertilizer industry, has shown potential in neutralizing cement and mitigating ASR due to its chemical composition [57]. Phosphogypsum primarily contains calcium sulfate (CaSO_4_) and other impurities such as phosphates, which can play a role in altering the cement matrix and reducing ASR. Phosphogypsum, when added to concrete mixtures, can reduce the overall alkalinity of the cement paste. ASR occurs when high pH levels (alkaline conditions) promote the reaction between alkali hydroxides in cement and reactive silica in aggregates. By incorporating phosphogypsum, the sulfate content can react with the alkali hydroxides, reducing the pH of the concrete matrix and thus lowering the potential for ASR to occur.

Despite the extensive research on ASR mitigation and the use of SCMs, there remain gaps in understanding the combined effects of different SCMs, particularly in binary and ternary blends, on both ASR mitigation and the overall mechanical properties of concrete. While previous studies have explored the use of materials such as metakaolin (MK), waste glass powder (GP), and other pozzolans, limited research has investigated their combined efficacy in reducing ASR expansion while maintaining concrete strength and workability. Furthermore, the use of new cementitious products like “NewCem Plus” and the incorporation of fibers such as basalt, alongside lithium-based treatments, have not been fully explored in combination with these SCMs. This paper addresses this gap by evaluating the effectiveness of binary SCM blends, including MK, GP, and NCM, as well as the roles of basalt fibers and lithium in ASR mitigation. Through a comprehensive experimental approach, including a 14-day accelerated mortar bar test (ASTM C1260) and assessments of compressive strength and workability, this study aims to provide a deeper understanding of the potential of these materials for improving the durability and performance of concrete, offering new insights for future applications.

## 2. Materials and Methods

The primary goal of this research was to investigate the effectiveness of different SCMs in mitigating ASR in concrete. The SCMs used in this study included MK, GP, basalt fibers, and lithium. To assess the influence of these SCMs on ASR expansion, the accelerated mortar bar test (ASTM C1260) was employed. A control mixture was prepared using 100% highly alkaline cement and fine reactive basalt aggregate without any SCMs. Experimental mixtures were then developed by incorporating high percentages of these SCMs, either as binary or ternary blends, with the aim of determining the optimal combination and dosage that most effectively inhibited ASR in concrete.

### 2.1. Materials and Design

**Figure 1:**
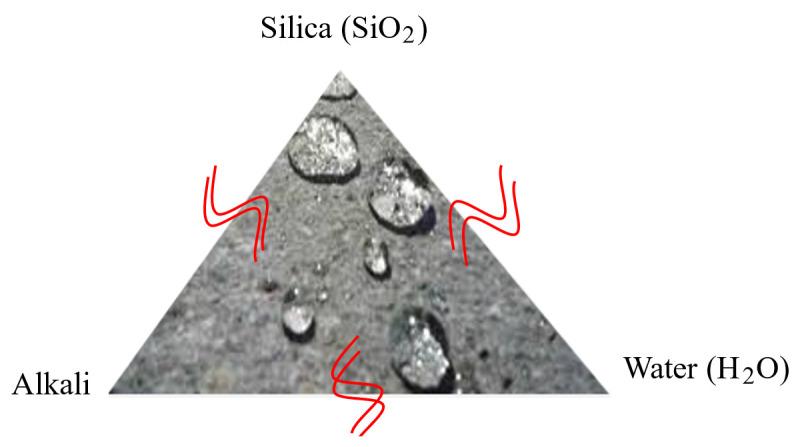
Alkali Silica Reaction Triangle (Concrete Cracking—A Destructive Kind).

The raw materials used in this research were carefully selected based on their chemical composition to achieve the desired reduction in ASR, as illustrated in Figure 1. In designing concrete mixtures, it is crucial to define non-reactive blends that disrupt the chemical interactions between alkali, silica, and water, which are responsible for initiating ASR. Portland cement, for instance, contains a high percentage of lime (CaO), which promotes the alkali reaction due to its lower content of amorphous silicates. This makes it advantageous to partially replace Portland cement with materials that have lower CaO content, thereby reducing the likelihood of ASR initiation [58].

Various supplementary cementitious materials (SCMs) were identified in this study for mitigating ASR. The materials considered included MK, GP, basalt fibers, NewCem Plus, and lithium admixture. These SCMs exhibit low CaO content and high SiO_2_ percentages, as detailed in Table 1. Additionally, they are cost-effective and suitable for large-scale integration into concrete, particularly since some, like waste GP, are by-products of other industrial processes. MK and GP are recognized as pozzolanic materials due to their fine particle size, averaging less than 100 μm, which facilitates a pozzolanic reaction. According to Glasser F.P. (1992), for a substance to be considered pozzolanic, it must possess three key characteristics: a high silicate content (SiO_2eq_ = SiO_2_ + Al_2_O_3_ + Fe_2_O_3_), an amorphous structure, and a large surface area. These characteristics are present in both MK and GP, along with their high alkalinity (pH > 12.5), as indicated in Table 1.

NewCem Plus is a relatively new cement product developed by Lafarge Inc. It is a blend of fly ash and ground granulated blast furnace slag (GGBFS), with specifications that exceed the requirements outlined in the ASTM C1697 for blended SCMs [59]. The chemical composition of NewCem Plus is presented in Table 1. This innovative blended cement offers superior performance in terms of its strength, compatibility with admixtures, and versatility in various concrete applications. It combines the benefits of pure Portland cement, fly ash, and slag, but its specific impact on mitigating ASR has not been previously explored. Generally, the use of SCMs tends to reduce the risk of ASR, as highlighted in the existing literature, though it may sometimes compromise the compressive strength of concrete [58]. To address this potential trade-off, lithium (Li) was incorporated as an additional chemical admixture in some of the mixtures in this study to assess its influence on both ASR mitigation and the compressive strength of the concrete.

Lithium is the second element in Group 1 of the periodic table, with an atomic mass of 6.941 g/mol and an atomic number of 3. As an alkali metal, lithium is highly reactive in its natural state due to its single valence electron in its outermost shell, which makes it prone to reacting with water and moisture. Commercially, stable lithium compounds are typically found in the forms of lithium carbonate (Li₂CO₃), sulfate, nitrate, and chloride. All alkali metals in Group 1 share similar chemical properties, forming analogous compounds due to their chemical similarities. For instance, they readily form salts like sodium chloride (NaCl) through exothermic reactions. Because of its high reactivity, lithium is not found in its elemental form in nature. Instead, it is commonly found in compounds such as lithium chloride in brine, which is easily dissolved. Additionally, the mineral spodumene (LiAlSi₂O₆) is a naturally abundant source of lithium.

Lithium’s primary commercial use is in the construction industry as an admixture for concrete. Due to its high reactivity, lithium interacts with the silicates and carbonates in concrete, lowering the alkalinity of the pore solution. This reaction forms unreactive compounds that help inhibit the ASR, which is triggered by the presence of moisture. In addition to its role in concrete, lithium is widely used in lithium-ion batteries, which power modern gadgets like laptops, smartwatches, cell phones, and tablets. The rising demand for lithium in these technologies has led to an increase in its production. The use of lithium in concrete mixtures has shown promise in reducing the harmful effects of ASR [49]. Lithium nitrate (LiNO₃) at a concentration of 30% is commonly used as an admixture to mitigate ASR. The required concentration of lithium depends on the nature of the concrete mixture, with reactive aggregate types needing higher lithium-ion concentrations to effectively reduce ASR. A lithium-to-sodium-and-potassium ratio of 0.74 has been found to eliminate ASR in the most reactive aggregates used in concrete [60]. This ratio, now a standard in the industry, requires 4.6 L of 30% lithium nitrate to be added for every 1 kg of sodium oxide present in a mixture [49].

**Table 1:** Chemical Compositions of Used SCMs [61,62].

Material	Chemical Composition	Percentage (%)	Particle Size
Portland Cement	CaO	60–67	7–200 μm
	SiO_2_	17–25	
	Al_2_O_3_	3–8	
	Fe_2_O_3_	0.5–6	
Metakaolin	SiO_2_	50–55	2 μm
	Al_2_O_3_	40–45	
Glass Powder	SiO_2_	68	200 μm
	CaO	14.5	
	K_2_O	0.8	
Basalt Fibers	SiO_2_	51–59	N/A
	Al_2_O_3_	14.6–18.3	
	CaO	5.9–9.4	
	MgO	3–5.3	
NewCem Plus	CaO	15	12.34 μm
	Other Alkali	2.5	
	SO_3_	3	

**Figure 2:**
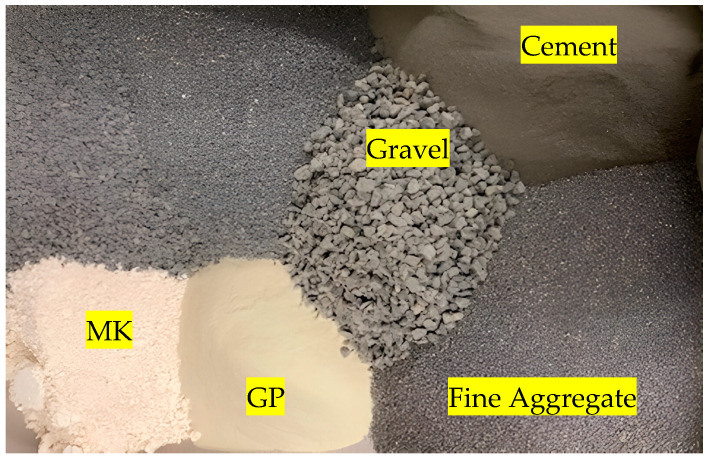
MK and GP Samples.

Firstly, a control concrete mixture was designed following the ASTM C1260 to achieve a 7500 psi compressive strength at 28 days. Then, a total of twenty-six different concrete mixtures were considered in this study, using type I Portland cement, according to the ASTM C150 [63]. The cement and water contents were 440 kg/m^3^ and 207 kg/m^3^, respectively. The water-to-cement ratio (w/c) was 0.47. The basalt aggregate used had five different particle gradings of 2.36 mm, 1.18 mm, 600 µm, 300 µm, and 15 µm. The twenty-six mixtures were prepared by replacing different replacement ratios (10%, 20%, and 30%) of cement with MK, GP, NCP, and fibers, as shown in Table 2. Figure 2 shows the raw materials in the MK and GP samples. The specific gravity (G) of the materials was used to determine the required quantity for replacement materials, where MK had a G of 2.30; GP had a G of 2.73; NCP had a G of 2.75; and the basalt fibers had a G of 2.65.

### 2.2. Testing Procedures

To evaluate the effectiveness of the ASR mitigation methods, various tests documented in the civil engineering literature were employed. Given that mitigation techniques differ across regions, assessing their effectiveness is crucial in confirming the overall viability of these methods. In this study, the primary test used was the ASTM C1260-7, commonly referred to as the accelerated mortar bar test (AMBT). This test measures the expansion of concrete due to ASR under accelerated environmental conditions, providing insight into the material’s susceptibility to ASR. The test matrix, outlining the specific mixture compositions, is presented in Table 3. This method allowed for a rapid assessment of ASR potential and the effectiveness of the different SCMs used in this research.

#### 2.2.1. Accelerated Mortar Bar Test

The accelerated mortar bar test (AMBT) was used to assess the effectiveness of the SCMs and glass powder in mitigating ASR. This test was conducted in accordance with the ASTM C1260 [17]. Mortar samples measuring 25 mm × 25 mm × 285 mm (1 in × 1 in × 11.25 in) were prepared and molded following the standard aggregate gradation outlined in the ASTM C1260 [64]. After molding, the samples were stored at room temperature with 95–100% relative humidity for 24 h. Following this initial curing, the specimens were demolded and cured in water at 80 °C for an additional 24 h. The samples were then fully submerged in a sodium hydroxide (NaOH) solution at a consistent temperature of 80 °C (176 °F) for 14 days. The expansion of the specimens was monitored and evaluated over this period, following the guidelines specified in the ASTM C1260 [65], to determine the effectiveness of the SCMs and glass powder in reducing ASR-induced expansion.

It is important to note that the 14-day AMBT was particularly suited for mortar samples containing fine aggregates, while the 56-day miniature concrete prism test (MCPT) is recommended for future research when evaluating coarse aggregate samples. In this study, as shown in Table 3, a total of twenty-six mortar mixtures containing various pozzolans were tested, with each mixture having at least three duplicates to ensure accuracy. Length measurements were recorded after 14 days. According to the test criteria, the pozzolans were considered effective at mitigating ASR if the expansion of the mortar samples after 14 days was less than 0.10% of any reactive aggregate. However, if the sample expansion exceeded 0.20%, the result was deemed ineffective and detrimental to the concrete’s durability.

In all mixtures, cement was partially replaced with varying percentages of additives or SCMs at the levels of 0%, 10%, 20%, and 30%. After the mortar bars were demolded, they were placed in a water bath for 24 h at a consistent temperature of 80 °F. Following this, the bars were immersed in a one molar sodium hydroxide (NaOH) solution for the duration of the test, which lasted between 7 and 14 days. At periodic intervals, the mortar bars were removed, and their length changes were measured to evaluate expansion. The expansion changes were calculated using Equation (1), and the results were recorded for further analysis.

An expansion of the mortar bars exceeding 0.13% indicated a failure to prevent the ASR in detrimentally reactive aggregates when exposed to the alkaline solution. To establish a safe level, the expansion threshold was reduced to 0.06%. The results from the 14-day AMBT were found to correlate well with the outcomes of the 1-year and 2-year concrete prism tests (CPTs). The formula used to calculate concrete expansion is presented below, where the percentage of length change in the test specimens at any given age (X days) was calculated as

(1) $$L(\%)=\frac{L_{x}-L_{i}}{G}\times 100$$

where L = Change in length at X days, %;

$L_{x}$ = Comparator reading of test prism at X days minus the comparator reading of the reference bar at X days;

$L_{i}$ = Comparator reading of test prism at zero day minus the comparator reading of the reference bar at zero day;

G = Nominal gauge length, 10 inches.

For the measurement of the reactivity of the concrete mixtures, Table 4 was used as a guideline. The 14-day expansion using AMBT criteria was used in this study, where the results showed non-reactive ingredients if the expansion stayed below 0.1%; where with a range of 0.1% to 0.3%, the reactivity was classified as moderate; and where anything above that was termed highly and very highly reactive (see Table 4).

The first batch of cement mixture was made up of the control mixture only; alkali cement and fine basalt aggregate were used. This mixture was used to establish a control case for the baseline test. The mixture was allowed to settle in the rectangular molds according to the AMBT using ASTM C1260 standards. The bars were immersed in one molar sodium hydroxide solution for 14 days, and data were collected at periodical intervals by measuring the bars’ length change. For this to be a successful baseline test, the concrete bars had to expand beyond the safety level of 0.1% in 14 days to ensure that the aggregate and the cement were producing sufficient ASR.

The next step was to include the SCMs in the cement mixtures one at a time to test their individual effect on the reactive elements inside the concrete mixture. The first group of mortar bars was produced by mixing the prescribed quantity of metakaolin with the cement and aggregates (Table 3). The mixtures were prepared by repeating the process with different waste glass powder replacements as in the control test.

The second group was made with GP with the same percentage of cement replacement. The third one was made by adding GP and MK at various percentages (binary mix) to study the combined effect of these two SCMs on the reactivity of aggregates used. The fourth group was prepared using NCP. The rest of the procedure was repeated similarly for the other SCMs with different percentages of cement replacements. The test results were tabulated and compared for the calculation of the result.

#### 2.2.2. Flow Test

**Figure 3:**
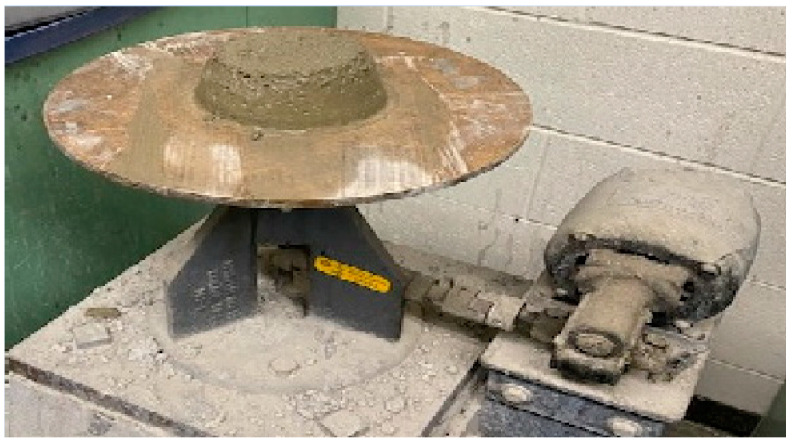
Flow Test.

The ASTM C1437 flow test [68] was used to measure the workability of the mortar samples (Figure 3). The workability or flow is the ease with which a mortar could be set. The mixture flowability test is important for ensuring adequate consolidation of test specimens, even if the workability performance by itself has no direct effect on ASR mitigation. This test involved filling a 4-inch steel cone in two levels with fresh mortar that was set in the center of a vibrating table. Twenty tamper rod strokes were used to compress each layer, and then the mixture was subjected to 25 drops applied in a 15 s period. Afterward, using Equation (2), the changes in the mortar sample diameter were measured from three different points to estimate the percentage flow of each combination.

(2) $$percent\;flow=\frac{D_{avg}-D_{o}}{D_{o}}\times 100$$

where

$D_{avg}$ = Average diameter size after 25 drops;

$D_{o}$ = Original diameter size (i.e., 4 inches).

#### 2.2.3. Strength Activity Index

In compliance with the ASTM C311 [69], the mortar sample Strength Activity Index (SAI) was evaluated. The pozzolanic activity of each mixture containing SCMs was evaluated and compared using this test for all binary and ternary combinations in the present research. In this experiment, two layers of new mortar were applied to a 2-inch steel cube, and each layer was crushed with 20 tamper rod strokes. At 28 days, a total of 26 mixtures including three replicates for each mixture were used, including different proportions of replacements for the Portland cement, as shown in Table 3. After that, the cubes were placed in a curing chamber with normal room temperature and humidity levels. The samples were demolded and left to cure at room temperature in a lime-saturated water tank after a 24 h period. After 7, 14, and 28 days, a 300 kip concrete compression machine (MC-300PR, Gilson Co., Lewis Center, OH, USA) was used to measure each sample’s compressive strength at a rate of 35 psi per second. Equation (2) was used to determine the SAI for each sample.

(3) $$strength\;activity\;index\;(SAI)=\frac{X}{Y}\times 100$$

where

X = average compressive strength of different mixtures ($N/mm^{2}$);

Y = average control compressive strength of mortar sample ($N/mm^{2}$).

## 3. Results and Discussion

### 3.1. Accelerated Mortar Bar Test

**Figure 4:**
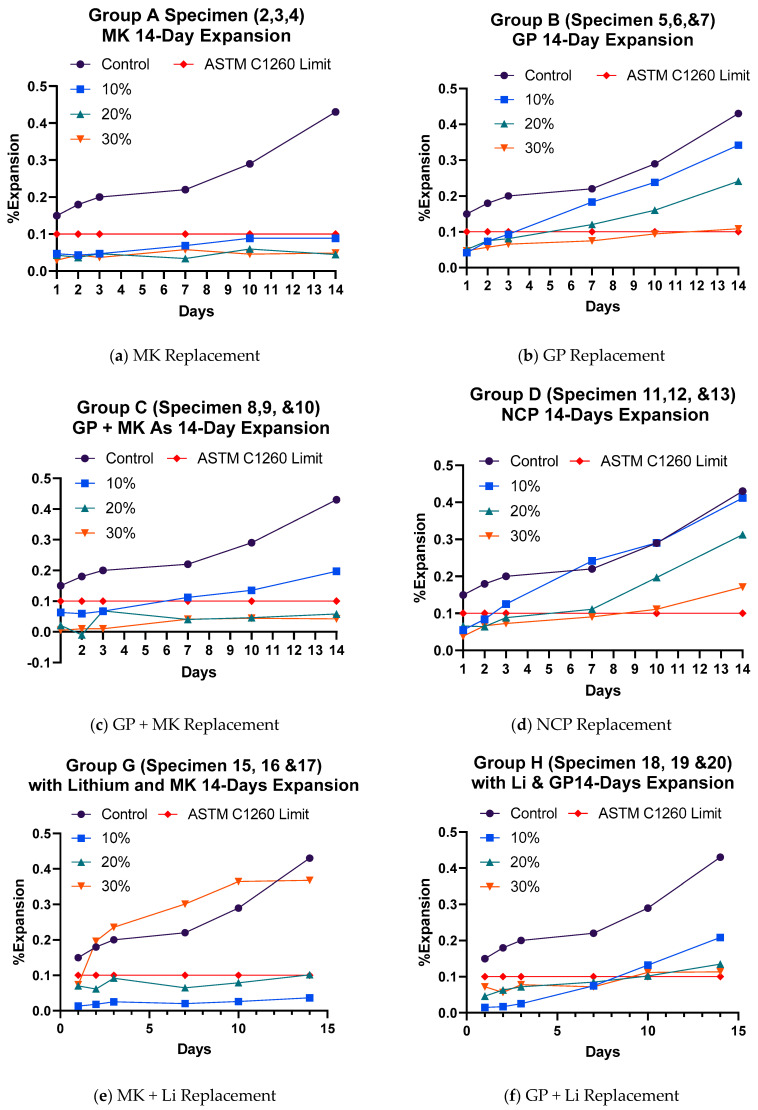

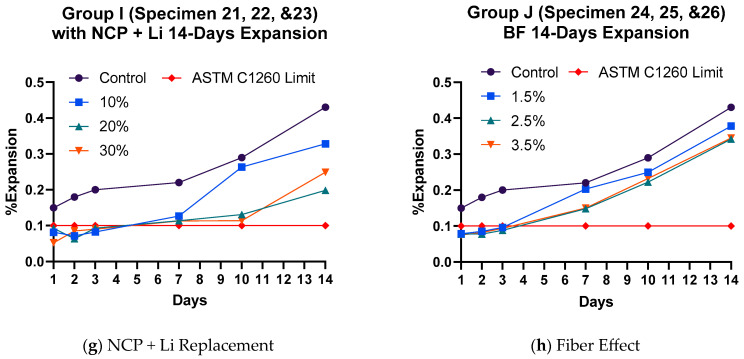
The 14-Day Accelerated Mortar Bar Expansion Rates.

**Figure 5:**
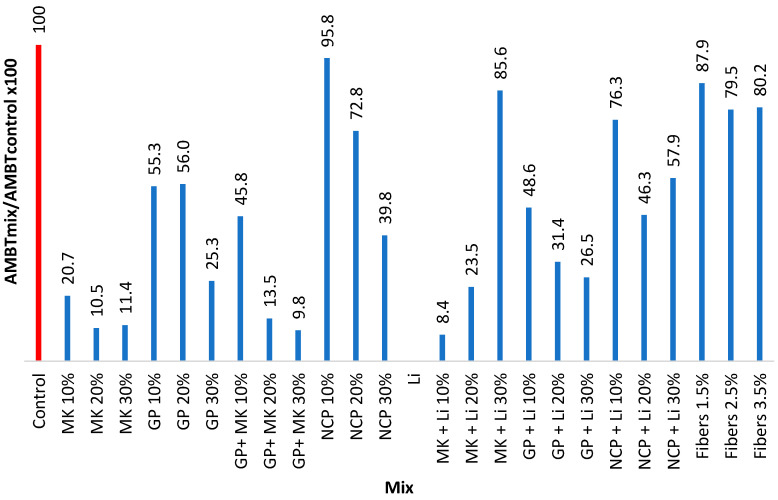
The 14-Day AMBT Normalized Expansion Rates.

The results of the accelerated mortar bar test are summarized in Table 5 and Figure 4 and Figure 5.

#### 3.1.1. Metakaolin Replacement

The results indicated that all samples with 10%, 20%, and 30% metakaolin (MK) cement replacement significantly reduced their ASR expansion, maintaining the concrete expansion below the ASTM safe limit of 0.10% (Table 5). This reduction in ASR expansion can be explained through the chemical properties of MK. MK is a pozzolanic material, rich in silica (SiO₂) and alumina (Al₂O₃), which reacts with calcium hydroxide (Ca(OH)₂) produced during the hydration of Portland cement. This pozzolanic reaction forms additional calcium silicate hydrate (C–S–H), the primary strength-giving compound in concrete, while simultaneously consuming calcium hydroxide. By reducing the availability of calcium hydroxide, MK lowers the alkalinity of the cement paste, which in turn minimizes the interaction between alkalis and reactive silica in aggregates, thus mitigating ASR.

In contrast, the control samples without MK exhibited a significant expansion of 0.425% due to the high concentration of free calcium hydroxide that promoted ASR (Figure 4a). The samples with 10%, 20%, and 30% MK replacement showed reductions in expansion by 79%, 89%, and 88%, respectively, compared to the control. This is because the pozzolanic reaction not only reduces alkali reactivity but also forms a denser concrete matrix, limiting moisture ingress, which is essential for ASR to occur.

#### 3.1.2. Glass Powder Replacement

GP was tested as an SCM at replacement levels of 10%, 20%, and 30% in Group B mixtures. All samples with GP replacement showed expansion percentages exceeding the ASTM threshold limit starting at three days (Figure 4b), but the expansion was still reduced compared to the control specimens by 20%, 43%, and 75% for 10%, 20%, and 30% replacement levels, respectively (Table 5). The mixture with 30% GP replacement slowed the formation of ASR gel around aggregate particles, with its expansion nearly falling below the ASTM C1260 limit. However, GP alone is classified as moderately reactive and is not recommended as a sole solution for mitigating ASR.

This limited effectiveness can be explained by the high silicate content in glass powder. Glass powder contains a significant amount of amorphous silica (SiO₂), which reacts with calcium hydroxide (Ca(OH)₂) generated during cement hydration. This reaction forms additional calcium silicate hydrate (C–S–H), which is beneficial for reducing ASR because it consumes Ca(OH)₂, a key component that increases the alkalinity of the concrete pore solution. However, at higher GP contents, the reaction can further reduce the alkalinity of the pore solution, limiting the availability of alkalis needed for the ASR. Despite this, the GP replacement alone does not fully prevent ASR because its pozzolanic reaction is slower than required to halt the formation of ASR gel completely. As a result, while glass powder reduces expansion and slows ASR progression, it is not as effective when used alone, due to the high silicate content still contributing to the reaction.

#### 3.1.3. Metakaolin and Glass Powder Replacement

Group C mixtures were binary combinations, incorporating both MK and GP to assess their combined effect on concrete. The results indicated a reduction in the alkali reactivity of aggregate particles, attributed to the presence of metakaolin, which was comparable to the reduction seen with metakaolin alone (Figure 4c). Mixtures with 20% and 30% MK/GP replacement ratios were effective at mitigating harmful ASR in concrete, showing significant reductions in expansion by 86% and 90%, respectively (Table 5). However, samples with a 10% MK/GP ratio were not effective at reducing ASR expansion. The binary mixtures demonstrated a stronger effect in mitigating ASR compared to individual mixtures containing either MK or GP alone.

This aligns with the findings from earlier research [70], which highlighted the synergistic benefits of using glass powder in binary and ternary blends for ASR mitigation. The study also found that a 20% replacement of SCMs, including glass powder, slag, or silica fume, effectively minimized ASR expansion without compromising other concrete properties. Furthermore, the ternary blends involving glass powder performed better than single SCMs, reinforcing the effectiveness of combining materials for enhanced ASR mitigation. Similar to the findings in this paper, the use of glass powder and SCMs reduced ASR expansion and was supported by microstructural analysis, which showed fewer cracks due to ASR expansion. The binary mixtures in this research confirm the importance of utilizing blended SCMs to achieve substantial ASR mitigation, with higher replacement ratios of MK/GP offering superior performance.

#### 3.1.4. NewCem Plus Replacement

NCP, an additive material for cement mixtures produced by Lafarge Industries (Jaipur, India), is designed to enhance the durability and mechanical properties of concrete. However, the tested mixtures with NCP did not sufficiently mitigate the ASR, with concrete expansion exceeding the ASTM C 1260 safety limit of 0.1%. Three mixtures were prepared using 10%, 20%, and 30% replacement of the total cement quantity with the NCP blend. The 14-day ASTM C 1260 test results for these mixtures, along with the control and the safety limit, are presented in Figure 4d. The 10% replacement showed no effect on ASR mitigation by the 14th day, while the 20% mixture reduced expansion in the concrete bars by 26% compared to the control mixture. The greatest reduction in expansion was observed in the 30% replacement sample, achieving around 60% less expansion than the control mixture. However, none of the samples were able to keep the expansion under the ASTM safety limit of 0.1%.

#### 3.1.5. Lithium Replacement

**Figure 6:**
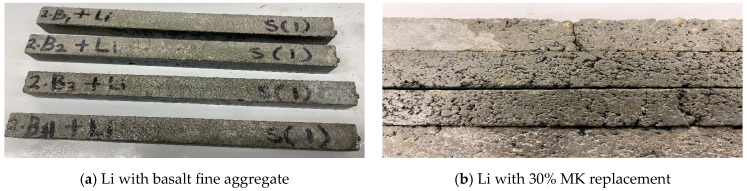

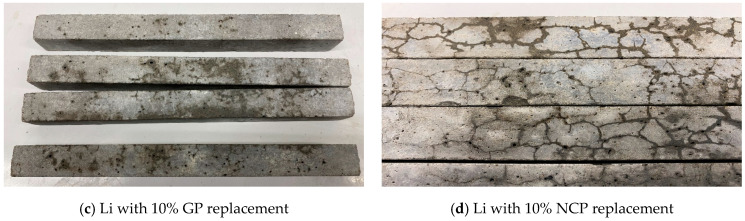
Damage Patterns of the AMPT of Lithium Specimens.

In the Group F tests, lithium was mixed with a basalt aggregate without adding SCMs to evaluate lithium’s ability to control expansion over time. As expected, the results in Figure 6a showed no signs of ASR cracks after 14 days under harsh conditions, as measured by the accelerated mortar bar test. This absence of expansion was due to the chemical interaction between the fine basalt aggregate and lithium.

When lithium was combined with MK in cement replacement ratios of 10%, 20%, and 30%, the 10% lithium and MK sample showed an excellent reduction in ASR expansion, with a 92% decrease, resulting in only 0.036% total expansion after 14 days (Figure 4e). This demonstrates that MK and lithium are highly effective at reducing expansion. However, the 20% lithium and MK mixture showed an increase in expansion compared to the 10% sample, with a total expansion of 0.1%, which is still within the ASTM C 1260 safety limit. On the other hand, the 30% replacement sample showed abnormal and erratic expansions, making it unsuitable for use in construction.

In another test, lithium was combined with GP, and the samples were immersed in NaOH solution for 14 days. The addition of GP and lithium successfully reduced ASR-related expansion. The 10% replacement resulted in 0.209% expansion (a 51% reduction), while the 20% replacement achieved a 68% reduction, and the 30% replacement further improved the reduction to 73% (Figure 4f). Despite the significant reductions, the 20% and 30% replacements did not pass the ASTM test, with total expansions of 0.209%, 0.135%, and 0.114%, all exceeding the 0.1% safe limit.

Lastly, three samples were made with a mixture of NCP and lithium. While the 14-day test results showed reduced expansion compared to untreated concrete, none of the samples passed the ASTM C 1260 test. The total expansion for the samples was 0.328%, 0.199%, and 0.249%, with the 20% replacement showing the best performance, reducing expansion by 53% compared to the control sample’s 0.43% expansion (Figure 4g). However, the 10% and 30% mixtures did not significantly mitigate ASR effects. As a result, the combination of NCP and lithium was not effective at reducing ASR expansion below the ASTM safe limit.

#### 3.1.6. Fiber Effect

The effect of the basalt microfibers (BFs) on ASR mitigation was tested using three mixtures containing 1.5%, 2.5%, and 3.5% of BFs, corresponding to 0.2 g, 0.3 g, and 0.4 g of fibers, respectively. The results, shown in Figure 4h, indicate that the addition of basalt fibers was not effective at preventing ASR, as all samples exceeded the safety threshold after three days.

The results demonstrated that the MK mixtures significantly reduced ASR expansion, showing a considerable improvement. Similarly, the Li mixtures performed as expected, with very low expansion rates. The combination of GP and MK at 20% and 30% replacement levels also showed promising results in mitigating ASR. However, mixtures containing NCP and basalt fibers exhibited higher expansion rates compared to the control mixture, indicating that these materials did not effectively mitigate ASR expansion.

### 3.2. Flow Test

The workability of all samples was tested according to the ASTM C1437 flow test. The flow percentage was calculated based on the average diameter, measured in two perpendicular directions of the inner mold. The control mixture exhibited a workability percentage of 103%, indicating excellent flow.

For Group A (MK mixtures), workability decreased as the percentage of MK replacement increased, which can be attributed to the angular shape of MK particles, as shown in Table 5. In Group B (GP mixtures), the workability was lower than the control mixture but improved as the GP replacement percentage increased. This improvement was due to the spherical nature of the GP particles, which created a rolling effect. This finding aligns with Ref. [61], who noted that the reduced cohesiveness between the impermeable glass powder and its lower absorption capacity contribute to the increased workability. For Group C (GP and MK mixtures) and Group D (NCP mixtures), the results indicated lower workability percentages compared to the control mixture. Additionally, as the replacement ratio increased, the workability further decreased, as shown in Table 5. This suggests that higher concentrations of cement replacements may have affected the practicality of the mixtures. Similarly, Group H (MK and Li mixtures) exhibited a reduction in workability. The added SCMs absorbed extra moisture from the concrete, making the mixture less workable. The 30% blend had particularly low workability, causing the mix to dry quickly, while the 10% blend showed workability comparable to the control mix. Lastly, for Group I (NCP and Li mixtures), the workability of the 20% and 30% cement replacement samples significantly decreased, while the 10% replacement sample showed no notable change in workability.

### 3.3. The 28-Day Compressive Strength Test

The results of the compression test after 28 days are presented in Table 6.

#### 3.3.1. Metakaolin Replacement

The 10% and 20% mixtures containing MK showed an increase in compressive strength compared to the control cubes at 28 days (Table 6). The inclusion of fine metakaolin particles enhanced the hydration process, leading to the production of additional C–S–H, which significantly contributed to the strength of concrete. The results across these MK samples demonstrated that concrete strength continued to increase with age, further indicating the positive impact of metakaolin on long-term strength development.

#### 3.3.2. Glass Powder Replacement

The compression test results revealed that the 10% GP sample exhibited compressive strength comparable to the control sample (see Table 6). However, when the GP replacement levels were increased to 20% and 30%, the compressive strength decreased significantly, reaching only 68% of the strength of the control mixture.

#### 3.3.3. Metakaolin and Glass Powder Replacement

The compressive test results for the binary mixtures of MK and GP, as shown in Table 6, revealed a reduction in compressive strength for the 20% and 30% MK/GP samples, with strengths of 81% and 84%, respectively, compared to the control. This suggests that higher concentrations of combined MK and GP in the SCM mixtures significantly weakened the concrete prisms.

#### 3.3.4. NewCem Plus Replacement

The sample made with 10% cement replacement with NCM did not negatively affect the concrete strength; it increased the compressive strength compared to the control mixture. However, the 20% and 30% samples significantly reduced the compressive strength to be at 80% and 63% of the control samples, respectively, as shown in Table 6. The addition of more than 10% NCM is not recommended because it adversely affects the compressive strength of concrete.

#### 3.3.5. Lithium Replacement

The samples containing 10% and 20% MK + Li showed compressive strengths comparable to the control concrete blocks. This can be attributed to lithium’s ability to inhibit the reaction between the alkali in cement and reactive aggregates. However, the sample with 30% replacement resulted in an 18% reduction in concrete strength, as shown in Table 6.

The 28-day results for the GP + Li mixtures are presented in Table 6. The samples with 10% cement replacement achieved a breaking strength of 57 MPa, surpassing the control sample’s strength of 55 MPa. This indicates that the addition of 10% lithium and GP improved the concrete strength by approximately 5.5% at 28 days. Conversely, cement replacement levels of 20% and 30% led to reductions in the concrete strength by 10% and 20%, respectively.

For the NCP + Li mixtures, the results (Table 6) showed that the 10% and 20% samples had compressive strengths similar to the control mixture. However, the 30% NCP + Li sample resulted in an 18% decrease in compressive strength, yielding a strength of 45 MPa.

#### 3.3.6. Fiber Effect

The compressive strength of the basalt fiber samples revealed that higher fiber concentrations led to a decrease in compressive strength compared to the control sample. This reduction in strength is likely due to the creation of air bubbles around the fibers during mixing, which increased air voids in the concrete microstructure, thereby reducing compressive strength. However, the first sample showed an increase in compressive strength at both the 14-day and 28-day marks.

Despite a reduction in compressive strength in some samples, the concrete remained suitable for various applications. Notably, normal-weight concrete used in structural elements typically has a compressive strength of 27–35 MPa. The lowest recorded strength from the tested mixtures was 33 MPa (from the sample with 2.5% fiber), which is still considered a strong concrete and can be implemented in many applications.

## 4. Conclusions

After comparing the results of the different tests, we could see that all the SCMs used in our experiments varied between either decreasing or increasing the mortar bar ASR expansion based on the SCM replacement level. Metakaolin was shown to be a great ASR mitigating agent by reducing the amount of ASR gel formed in concrete. Metakaolin, which has pozzolanic properties, was mixed in different ratios as a cement replacement. When metakaolin was used at a 10% replacement, it completely mitigated the harmful effects of ASR in concrete by showing a significant reduction in the concrete expansion below the 0.1% threshold level. However, metakaolin reduced the flow of concrete and slightly reduced the compressive strength at 7 and 14 days. Microparticles of waste glass powder did not show positive results in the mitigation of ASR in concrete. The mortar bars made with these particles showed no resistance to changes in length and crossed the safety thresholds. Basalt fiber did not show promising results in mitigating the harmful effects of ASR in concrete. All samples went straight into expansion without delay. Moreover, the basalt fibers did not affect the concrete compressive strength. The combination of waste glass powder and metakaolin showed good positive results. The concrete showed increased resistance to alkalinity and increases in compressive strength. The ASR mitigation was effective at concentrations of 20% or more. The 10% cement replacement was not that effective at stopping the expansion of concrete. Overall, the method used for testing, the AMBT C1260, was an effective, quick measure of the effectivity of the reactivity of aggregate particles and the highly alkaline concrete mixtures. The specific conclusions are the following:

- In the samples where 10%, 20%, and 30% metakaolin was added, the expansion was 79%, 89%, and 88% less, respectively, than that of the control specimen.
- The GP decreased the expansion compared to the control specimens by 20%, 43%, and 75% at the 10%, 20%, and 30% replacement levels, respectively. It can be concluded that GP of a 30% replacement level is recommended.
- Mixtures 9 and 10 of MK + GP showed a significant reduction in ASR by 86% and 90% for the 20% and 30% replacement levels, respectively.
- The binary mixtures of GP + MK at the 20% and 30% levels were more effective than the individual mixtures of MK or GP in reducing the ASR below the threshold limit. The mixtures were effective in decreasing the ASR expansion (60% reduction) compared to the control mixture at 10% replacement.
- The sample with 10% lithium and MK showed an excellent reduction in the expansion caused by ASR (92% reduction). The 14-day results showed that the total expansion was almost 0.1%, which is within the safe condition of the ASTM C 1260 test.
- The cement replacement of 10% with GP + lithium resulted in 0.209% expansion (51% reduction). However, the 20% and 30% reductions were not enough to pass the ASTM test, and the total expansion went above the 0.1% total expansion safe limit of the test.
- Therefore, the mixture of NCM and lithium was not an effective solution for ASR reduction in concrete.
- The results showed that the basalt fibers did not help in stopping ASR in concrete. After the third day, all samples had crossed the safety threshold of 0.10% expansion, and the results were very comparable to the control mixtures.

Based on the results of this study, we suggest using more than one SCM in concrete to make it more resistant to varying parameters. More research is needed to reach reliable and specific results for the concrete mixtures, especially with the continuous demand for concrete mixes. Using manufacturing materials in future research can support concrete’s durability and help achieve better solutions for mitigating ASR. The implementation of waste by-product such as MK and GP and additives like lithium has become more crucial for inhibiting the propagation of ASR, which will save billions of dollars that are spent in infrastructure rehabilitation.

## Figures and Tables

**Table 2:** Details of the Mix Design.

	Description		Fine Aggregate (Basalt) (kg/m^3^)					Water (lit)	Cement (kg/m^3^)	Admixtures (NaOH) (kg/m^3^)	MK (kg/m^3^)	GP (kg/m^3^)	NCP (kg/m^3^)	Li (lit)	Basalt Fibers (kg/m^3^)
MIX	Description	Cement Replacement (%)	2.36 mm	1.18 mm	0.600 mm	0.300 mm	0.150 mm								
1	Control	0%	124	235	235	235	161	207	440	4.37	0	0	0	0	0
2	MK	10%	124	235	235	235	161	207	396	4.37	32.12	0	0	0	0
3	MK	20%	124	235	235	235	161	207	352	4.37	64.25	0	0	0	0
4	MK	30%	124	235	235	235	161	207	308	4.37	96.36	0	0	0	0
5	GP	10%	124	235	235	235	161	207	396	4.37	0	38.13	0	0	0
6	GP	20%	124	235	235	235	161	207	352	4.37	0	76.26	0	0	0
7	GP	30%	124	235	235	235	161	207	308	4.37	0	114.40	0	0	0
8	GP + MK	10%	124	235	235	235	161	207	396	4.37	16.06	19.06	0	0	0
9	GP + MK	20%	124	235	235	235	161	207	352	4.37	32.125	38.13	0	0	0
10	GP + MK	30%	124	235	235	235	161	207	308	4.37	48.18	57.20	0	0	0
11	NCP	10%	124	235	235	235	161	207	396	4.37	0	0	38.41	0	0
12	NCP	20%	124	235	235	235	161	207	352	4.37	0	0	76.82	0	0
13	NCP	30%	124	235	235	235	161	207	308	4.37	0	0	115.24	0	0
14	Li	0%	124	235	235	235	161	207	440	4.37	0	0	0	0.130	0
15	MK + Li	10%	124	235	235	235	161	207	396	4.37	32.12	0	0	0.130	0
16	MK + Li	20%	124	235	235	235	161	207	352	4.37	64.25	0	0	0.130	0
17	MK + Li	30%	124	235	235	235	161	207	308	4.37	96.36	0	0	0.130	0
18	GP + Li	10%	124	235	235	235	161	207	396	4.37	0	38.13	0	0.130	0
19	GP + Li	20%	124	235	235	235	161	207	352	4.37	0	76.26	0	0.130	0
20	GP + Li	30%	124	235	235	235	161	207	308	4.37	0	114.40	0	0.130	0
21	NCP + Li	10%	124	235	235	235	161	207	396	4.37	0	0	38.41	0.130	0
22	NCP + Li	20%	124	235	235	235	161	207	352	4.37	0	0	76.82	0.130	0
23	NCP + Li	30%	124	235	235	235	161	207	308	4.37	0	0	115.24	0.130	0
24	Fibers	1.5%	124	235	235	235	161	207	440	4.37	0	0	0	0	5.55
25	Fibers	2.5%	124	235	235	235	161	207	440	4.37	0	0	0	0	11.10
26	Fibers	3.5%	124	235	235	235	161	207	440	4.37	0	0	0	0	16.65

180 g of NaOH; 4050 mL of water; and 270 mL of distilled water were used for the soak solution.

**Table 3:** Experimental Program.

Group	Mix No.	Variables	Replacement	Mortar Bars	Compression Test Cubes			Workability Base Flow Test
			(%)		7 d	14 d	28 d	
A	1	100% Cement	0	4	3	3	3	Once/batch
	2	MK	10	4	3	3	3	
	3	MK	20	4	3	3	3	
	4	MK	30	4	3	3	3	
B	5	GP	10	4	3	3	3	Once/batch
	6	GP	20	4	3	3	3	
	7	GP	30	4	3	3	3	
C	8	GP + MK	10	4	3	3	3	Once/batch
	9	GP + MK	20	4	3	3	3	
	10	GP + MK	30	4	3	3	3	
D	11	NCP	10	4	3	3	3	Once/batch
	12	NCP	20	4	3	3	3	
	13	NCP	30	4	3	3	3	
F	14	Li	N/A	4	3	3	3	Once/batch
G	15	MK + Li	10	4	3	3	3	Once/batch
	16	MK + Li	20	4	3	3	3	
	17	MK + Li	30	4	3	3	3	
H	18	GP + Li	10	4	3	3	3	Once/batch
	19	GP + Li	20	4	3	3	3	
	20	GP + Li	30	4	3	3	3	
I	21	NCP + Li	10	4	3	3	3	Once/batch
	22	NCP + Li	20	4	3	3	3	
	23	NCP + Li	30	4	3	3	3	
J	24	Fibers	1.5% volume fraction	4	3	3	3	Once/batch
	25	Fibers	2.5% volume fraction	4	3	3	3	
	26	Fibers	3.5% volume fraction	4	3	3	3	
Total				112	84	84	84	

**Table 4:** Reactivity Tables [66,67].

Reactivity	One-Year Expansion in CPT %	14-Day Expansion in AMBT %	56-Day Expansion in MCPT %
R0 Non-Reactive	≤0.04	≤0.10	≤0.03
R00 Slowly/Less Reactive	N/A	N/A	>0.031, ≤0.040
R1 Moderately Reactive	>0.04, ≤0.12	>0.10, ≤0.30	>0.041, ≤0.012
R2 Highly Reactive	>0.12, ≤0.24	>0.30, ≤0.45	>0.121, ≤0.240
R3 Very Highly Reactive	>0.24	>0.45	>0.241

**Table 5:** Accelerated Bar Mortar Test and Flow Test Results.

Group	Mix No.	Variables	Replacement	Average Expansion	Standard Error	Coefficient of Variation	Flow Test Average Diameter
			(%)	14 d (%)	(14 d)	(%)	(cm)
A	1	100% Cement	0	0.425	0.011	4.51	20.5
	2	MK	10	0.0890	0.0024	2.64	6.40
	3	MK	20	0.0450	0.0042	9.43	5.82
	4	MK	30	0.0490	0.0019	3.82	4.75
B	5	GP	10	0.342	0.0074	2.17	18.00
	6	GP	20	0.241	0.0035	1.47	19.00
	7	GP	30	0.108	0.0012	1.15	19.30
C	8	GP+ MK	10	0.197	0.0048	2.42	17.70
	9	GP+ MK	20	0.058	0.028	48.6	19.00
	10	GP+ MK	30	0.042	0.042	0.01	19.30
D	11	NCP	10	0.412	0.012	3.09	16.50
	12	NCP	20	0.313	0.018	5.93	11.40
	13	NCP	30	0.171	0.018	10.59	10.70
F	14	Li	N/A	N/A	N/A	N/A	N/A
G	15	MK + Li	10	0.036	0.0021	1.96	17.20
	16	MK + Li	20	0.101	0.0031	3.10	14.00
	17	MK + Li	30	0.368	0.0243	6.61	10.70
H	18	GP + Li	10	0.209	0.0012	0.57	20.80
	19	GP + Li	20	0.135	0.0035	2.58	17.20
	20	GP + Li	30	0.114	0.0027	2.36	14.30
I	21	NCP + Li	10	0.328	0.0035	1.06	20.30
	22	NCP + Li	20	0.199	0.0017	0.83	13.90
	23	NCP + Li	30	0.249	0.0012	0.50	11.40
J	24	Fibers	1.5% volume fraction	0.378	0.0104	2.74	18.70
	25	Fibers	2.5% volume fraction	0.342	0.0076	2.22	18.30
	26	Fibers	3.5% volume fraction	0.345	0.0037	1.06	19.00

**Table 6:** The 28-Day Compressive Strength Results.

Group	Mix No.	Variables	Replacement	Compressive Strength (MPa)	SAI
			(%)	(28 d)	(%)
A	1	100% Cement	0	55	100
	2	MK	10	61	112
	3	MK	20	64	117
	4	MK	30	53	96
B	5	GP	10	55	100
	6	GP	20	38	68
	7	GP	30	38	68
C	8	GP + MK	10	50	92
	9	GP + MK	20	45	81
	10	GP + MK	30	46	84
D	11	NCP	10	60	109
	12	NCP	20	44	80
	13	NCP	30	35	63
F	14	Li	N/A	58	106
G	15	MK + Li	10	54	99
	16	MK + Li	20	46	83
	17	MK + Li	30	58	105
H	18	GP + Li	10	50	90
	19	GP + Li	20	44	80
	20	GP + Li	30	58	106
I	21	NCP + Li	10	54	99
	22	NCP + Li	20	46	83
	23	NCP + Li	30	59	107
J	24	Fibers	1.5% volume fraction	34	62
	25	Fibers	2.5% volume fraction	40	72
	26	Fibers	3.5% volume fraction	55	100

## Data Availability

The original contributions presented in the study are included in the article, further inquiries can be directed to the corresponding author.
